# HER-3 Knocking Down Induces G2/M Arrest in Gastric Cancer Cells

**Published:** 2018

**Authors:** Ehsan Mokhtari, Hesamodin Mokhtari, Elham Moslemi

**Affiliations:** 1. Department of Biology, Faculty of Biological Sciences, Islamic Azad University, East Tehran Branch, Tehran, Iran; 2. International Campus, Shahid Sadoughi University of Medical Sciences, Yazd, Iran

**Keywords:** Epidermal growth factor receptor, Gastric adenocarcinoma, HER-3, Iran

## Abstract

**Background:**

The Human Epidermal growth factor Receptor-3 (HER-3) is a member of ErbB receptor family and has deficient kinase activity. HER-3 should heterodimerize with other members of ErbB receptor family, especially with HER-2, to transduce downstream signaling pathways. HER-3 co-expresses with other ErbB receptors in different cancers and overexpresses while the oncogenic signaling pathways such as Jak/Stat, MAPK, and PI3K/Akt are activated and promoted. Here, the expression level of HER-3 was evaluated in Iranian gastric adenocarcinoma’s patients and the effects of HER-3 knocking down was investigated on cell cycle and cell viability of human gastric adenocarcinoma cell line of MKN45.

**Methods:**

In this study, 38 paraffin-embedded surgical adenocarcinoma specimens and their marginal non-tumor tissue samples were collected. Total RNAs were extracted and cDNAs were synthesized. Finally, the expression level of HER-3 was evaluated by real time PCR approach. Moreover, the human adenocarcinoma cell line of MKN45 was transfected with siRNA against HER-3 and the effects of its down-regulation were evaluated using MTT assay and cell-cycle analysis.

**Results:**

The data obtained from this study revealed HER-3 is significantly overexpressed in gastric tumors rather than non-tumor marginal tissues. Also, it was found that the expression level of HER-3 is elevated with tumor depth of invasion. Moreover, HER-3 knocking down promotes cell accumulation in G2/M phase of cell cycle and decreases cell viability in MKN45 cells which suggests a potential role for HER-3 in gastric adenocarcinoma tumorigenesis.

**Conclusion:**

Taken together, these results emphasize the importance of HER-3 receptor in diagnosis and prognosis of gastric adenocarcinoma.

## Introduction

The human Gastric Cancer (GC) is the fourth most commonly diagnosed cancer and the second most common cause of cancer related death worldwide 1,2. Gastric carcinogenesis has been known to be a multistep and multifactorial process. Recent advances in molecular medicine have not only shed light on the carcinogenesis of gastric cancer, but also offered novel approaches regarding prevention, diagnosis and therapeutic intervention 3. Recently, HER-3, a member of receptor tyrosine kinases, has been introduced as a new biomarker in diagnosis and prognosis of gastric cancer in human 4.

The human Epidermal Growth Factor Receptor (EGFR) is the most studied family of Receptor Tyrosine Kinases (RTKs) that have important roles in signal transduction and carcinogenesis 5. ErbB receptors are transmembrane receptors consisting of four proteins including HER1/EGFR/ErbB1, HER2/ NEU/ErbB2, HER-3/HER-3 and HER4/ErbB4 6.

Two mechanisms promote receptor dimerization including direct binding of ligands and high receptor density due to overexpression 7. There is a large number of EGFR ligands that drive the receptor homo- or heterodimerization 8. HER-3 lacks intrinsic tyrosine kinase activity and it is unable to trigger downstream signaling pathways on its own 9. Therefore, HER-3 dimerizes with other ErbB receptors, specifically with HER-2 to become activated 10.

Of all the dimers formed by members of the ErbB receptor family, HER-2/HER-3 heterodimers are the most preferable activators of downstream signaling 10,11. The importance of HER-2 receptor in some cancers such as breast and gastric cancers has been verified in a lot of recent studies, whereas there are few reports focused on HER-3 as a significant factor involved in carcinogenesis 12. HER-3 is widely expressed in brain, prostate, kidney, lung, spinal cord and mammary glands and its expression is about 50% higher in cancer cells in comparison with normal cells 13. There are new evidences indicating that HER-3 overexpression is related to tumor size and increased metastasis 14. New researches focused on molecular mechanisms of function of HER-3 in carcinogenesis 13. The elevated expression of HER-3 confers chemo drug resistance in HER-2+ breast cancer cells through activation of PI-3 K/Akt signaling pathway 15,16. All of the mentioned evidences imply the important role of HER-3 receptor in tumor initiation and progression, and also chemo-resistance 14. Some recent studies considered that HER-3 is highly expressed in gastric cancer. It is believed that inhibition of HER-3 signaling with new recombinant drugs may be necessary to overcome chemo-resistance and efficient cancer treatment 17. Therefore, considering the importance of HER-3 in tumorigenesis, an attempt was made to evaluate the expression level of *HER-3* gene in Iranian populations suffering from gastric cancer. In addition, HER-3 was knocked down by siRNA in human gastric cancer cell line of MKN45 and its effects on cell cycle and cell viability were evaluated by flow cytometry and MTT assay, respectively.

## Materials and Methods

### Clinical samples collection

Human tumor and marginal non-tumor (normal tissues obtained from the margin of same tumors were used as controls) paraffin-embedded surgical specimens from 38 patients with gastric cancer specimens were kindly provided by Masoud Pathobiology Laboratory (Tehran, Iran). Twenty-six samples belonged to males and twelve samples were for females. Patients’ ages were in the range between 31 to 73 years old with average age of 54. According to tumor location, 22 samples were non-cardia and 16 samples were cardia tumor and based on grade of differentiation, the low, intermediate and high grades were 11, 13, and 14, respectively (Table 1). The stages and grades were confirmed with histopathological parameters according to WHO criteria.

**Table 1.** Pathological characteristics of patient’s sample

****Clinical characteristic****	****Number of patients****
****All patients****	38
****Gender****	
**Male**	26
**Female**	12
**Age (range)**	31–73 (51)
****Depth of invasion****	
**T1–T2**	11
**T3–T4**	27
****Grade of differentiation****	
**Low**	11
**Intermediate**	13
**High**	14
****Tumor location****	
**Cardia**	16
**Non-cardia**	22

### Cell culture

MKN45, a human gastric adenocarcinoma cell line, was provided by Pasteur Research Institute of Tehran, Iran. Cells were cultured in Dulbecco’s modified Eagle medium (DMEM) supplemented with 10% fetal calf serum, 100 *U/ml* penicillin and 100 *U/ml* streptomycin (Gibico, CA) and incubated at 37*°C* with 5% CO_2_.

### RNA extraction and cDNA synthesis

4 to 6 sections (each section in the size of 10 *μm*) were prepared for each block of paraffinized specimen by microtome. Xylene and alcohol were used for de-paraffinization and tissues were treated with proteinase K (Fermentase, Lithuania). Each pellet was separately treated with RNX plus solution (CinnaGen, Iran) according to the instruction of manufacturer. Quality and quantity of extracted RNAs were evaluated by UV spectrophotometry (260/280 *nm* ratio). RNAs were treated with DNaseI (Fermentase, Lithuania) to eliminate any unwanted DNA contamination. The first strand of cDNAs was synthesized using the reverse transcription system (TaKaRa, Japan). Non-reverse transcription (No-RT) control samples were acquired simultaneously from DNase treated RNA to find out any potential genomic DNA contamination.

### siRNA and cell treatment

*HER-3* siRNA duplexes were synthesized by Qiagen (Valencia, CA, USA). The targeted sequences (sense strand) were as follows: *HER-3*: 5′-AACCAATACCA GACACTGTAC-3′. For most experiments, approximately 2×10^5^ cells were plated in six-well plates in medium containing 10% FCS and cultured for 24 *hr* and then 75% confluence cells were transfected with siRNA using Lipofectamine 2000 (Invitrogen, Paisley, UK) as recommended by manufacturer’s instruction. The cells were harvested 0, 24, 48, and 72 *hr* after transfection by siRNA.

### Real-time PCR assay

The expression level of *HER-3* gene in tumor and marginal non-tumor samples was investigated by specific HER-3 primers: F; 5′-AGTGAGGCCAAGAC TCCAAT-3′, R; 5′-ACTCCCAAACTGTCACACCA-3′. Also, *GAPDH* gene was used as an internal control and its primer sequences were as follows: GAPF; 5′- A TGGAGAAGGCTGGGGCT-3′and GAP R; 5′-ATCT TGAGGCTGTTGTCATACTTCTC-3′.

Quantitative real time PCR was performed with SYBR green master mix based on manufacturer’s instruction. The PCR program was as follows: initial denaturation at 95*°C* for 10 *min*, followed by 40 cycles of denaturation at 95*°C* for 10 *s*, and annealing/ extension at 60*°C* for 30 *s*. The PCR product size verified by running on agarose gel and the authenticity of amplified fragment were confirmed by direct sequencing of PCR product.

### MTT assay

MNK45 cells were seeded at a density of 4×10^3^ cells/well on a 96-well plate. After transfection with siRNA against HER-3 and scrambling, viability of cells was investigated after 48, 72 and 96 *hr* by MTT assay. MTT [3-(4,5-dimethylthiazol-2-yl)-2,5-diphe-nyltetrazolium bromide] was added to each well at a concentration of 500 *μg/ml*, and plates were incubated for 4 *hr* at 37*°C*. Then, supernatants were removed and cells were lysed with 400 *μl* DMSO. Cells were incubated further for 10 *min* at 37*°C* with gentle shaking. Absorbance was measured at 570 *nM* using a computer-controlled microplate analyzer.

### Cell cycle analysis

Cells were seeded in 6 well plates with density of 2×10^5^ cells/well and incubated at 37*°C* overnight. Then, cells were transfected with *HER-3* siRNA, scrambled and incubated for 48 *hr*. Transfected cells were harvested and fixed by 70% cold ethanol at 4*°C* overnight and further analyzed by flow cytometry using PI/RNase staining buffer for cell cycle analysis. Distribution of cells in various cell phases was analyzed by Flowing Software 2.5.1.

### Western blotting analysis

After siRNA treatment, MNK45 cells were lysed in 1 *ml* lysis buffer composed of 50 *mM* Tris-HCL, pH=7.4, 5 *mM* EDTA, 1% Triton X-100, 150 *mM* NaCl and 1% protease inhibitor cocktail for 1 *hr* on ice with 15 *min* interval of vortexing for 30 *s*. Using protein assay kit (Bio-Rad Laboratories, CA), the protein concentrations were measured. 30 *μg* of cell lysates were used for SDS-PAGE. Then, resolved proteins were transferred onto PVDF membrane. Blocking was performed for overnight at 4*°C* with 5% non-fat milk in PBS plus 0.05% Tween 20. After three times of washing with PBS-Tween with 3% non-fat milk, PVDF membrane was incubated with 5 *μg* of anti-HER-3 monoclonal antibody (Abcam, Cambridge, UK) for 1 *hr* at room temperature. After three times of washing with PBS-Tween, membrane was incubated with Horseradish Peroxidase (HRP)- conjugated beta-actin antibody (Abcam, Cambridge, UK) for 1 *hr* at room temperature followed by washing and developing with ECL chemiluminescence detection system (Pierce ECL Substrate Western blot detection system, Thermo Scientific, IL) and was exposed to autoradiography film (Kodak XAR film).

### Statistical analysis

To calculate relative expression of HER-3 in patients, 2^−ΔΔCt^ formula was used. Differences in gene expression between two groups in each analysis were evaluated using one-sample t-test which was performed by SPSS ver. 20 and histograms drawn using Graph Pad Prism 6 Demo software package. The results are expressed as mean±SE of at least triplicate independent experiments. Comparisons between two groups were performed by Mann-Whitney U test. The comparison of HER-3 expression between various clinical characteristics was analyzed by the chi-square test. A p-value less than 0.05 was considered statistically significant.

## Results

### HER-3 is overexpressed in human gastric adenocarcinoma

**Figure 1.**
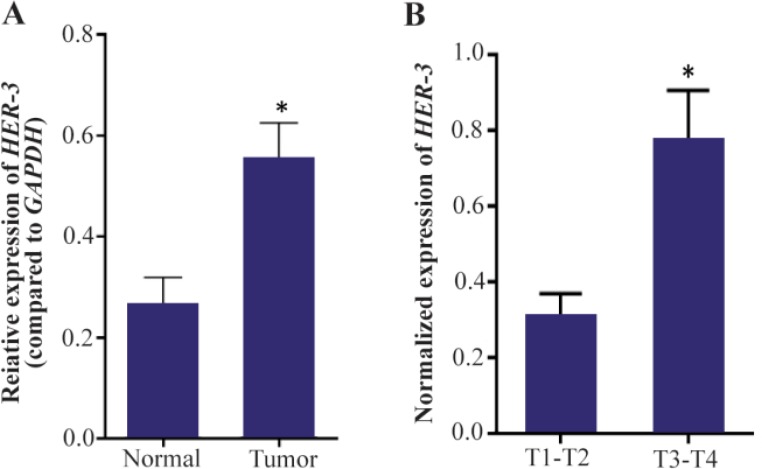
Real time PCR analysis of *HER-3*. The expression of *HER-3* is up-regulated in gastric tumors compared to the non-tumor marginal samples (p-value=0.01). Moreover, the expression level of *HER-3* gene is elevated with tumor depth in gastric adenocarcinoma (p=0.023). Real-time PCR was performed as duplicates for three times for each sample. The expression values were normalized relative to the expression level of *GAPDH* as a housekeeping gene.

Using specific primers, the expression level of *HER-3* was evaluated in tumor and non-tumor marginal tissues and between different grades of gastric adenocarcinoma. The results of this study manifested that HER-3 is expressed in both tumor and non-tumor marginal samples of stomach. However, the level of expression is significantly higher in tumor samples compared to non-tumor marginal samples (p-value=0.01). Moreover, in the tumor group, HER-3 expression was significantly associated with depth of invasion (p-value= 0.023) (Figure 1).

### HER-3 RNA and protein levels reduced after transfection of siRNA

As described in materials and methods, MKN45 cells were transfected with siRNA against HER-3 and scrambled control. After 72 *hr*, *HER-3* mRNA level was decreased about 80% compared to the scrambled control. Also, it was found that HER-3 protein level was sharply reduced in siRNA transfected cells.

### Suppressing HER-3 by siRNA arrested MKN45 cells in G2/M phase of cell cycle

**Figure 2.**
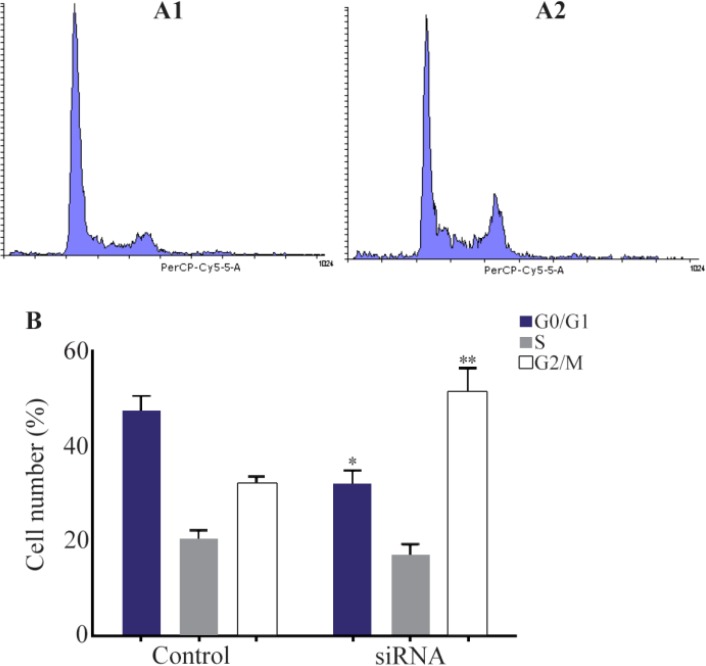
Cell cycle analysis of MKN45 cell lines using flow cytomtery. MNK45 cells were treated with *HER-3* siRNA (A1), scrambled (A2) and analyzed by flow cytometry after 48 *hr*. B) number of cells distributed in different cell cycle phases in cells transfected with siRNA and scrambled. *HER-3* silencing increased cell accumulation in G2/M phase of cell cycle up to 20% compared to the scrambled-treated control cells (p= 0.029).

Gastric adenocarcinoma cell line of MKN45 was transfected with siRNA against HER-3 and scrambled control. After staining cells with PI, distribution of cells in various phases of cell cycle was analyzed by flow cytometry. Our data revealed that cells treated with siRNA were accumulated in G2/M phase of cell cycle in comparison with the scrambled control cells. Also, the number of cells in G1 and S phases was decreased in siRNA treated cells (Figure 2).

### HER-3 down-regulation by siRNA decreased viability of MKN45 cells

**Figure 3.**
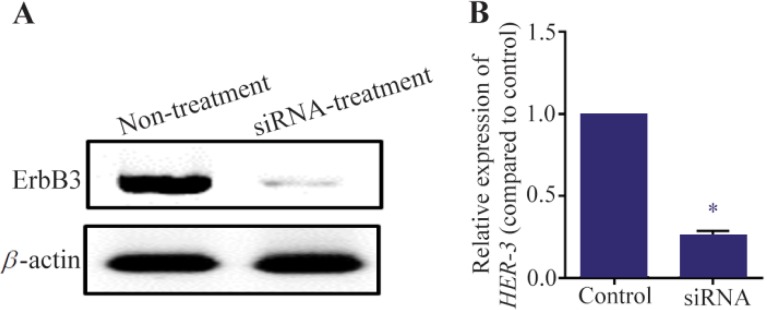
Western blotting assay for MKN45 cells. MKN45 cells were transfected with *HER-3* siRNAs and harvested 72 *hr* after transfection. The cells were lysed and 50 *mg* of total protein was used for detection of ErbB3 by western blotting. HER-3 protein level was decreased after treatment with siRNA. Beta-actin was used as internal control of western blot.

**Figure 4.**
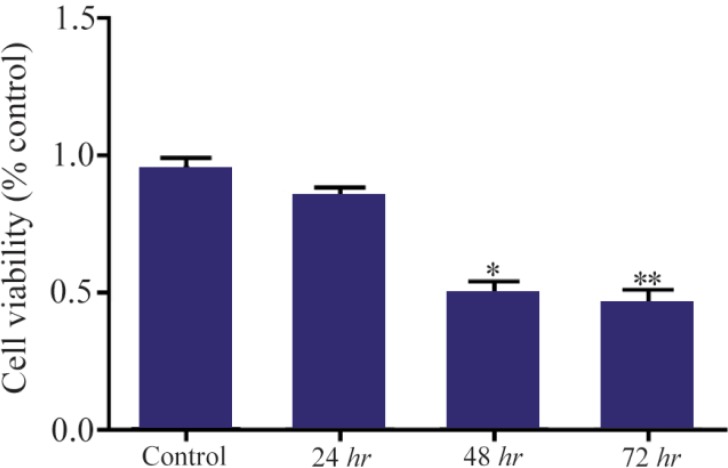
Cell viability analysis of siRNA treated MKN45 cells by MTT assay. MKN45 cells were harvested 24, 48 and 72 *hr* after ErbB3 siRNA transfection and analyzed by MTT assay. Our results showed that ErbB3 downregulation reduced cell viability by 50%, 48 *hr* after treatment with siRNA compared to scrambled control (p-value=0.013).

MKN45 cells were transfected with *HER-3* siRNA and prepared for MTT assay after 24, 48 and 72 *hr*. Our data showed that *HER-3* siRNA significantly decreased MKN45 cell proliferation by 50% after 48 hours (Figures 3 and 4).

## Discussion

Gastric adenocarcinoma is an aggressive cancer and remains the fourth most common type of cancer and is the second leading cause of cancer-related death worldwide. Gastric cancer is often diagnosed at an advanced stage. The most common therapy is surgical resection in company with chemotherapy or chemo-radiation in appropriate cases 18. Unfortunately, treatment of advanced or metastatic gastric cancer has seen little progress and median Overall Survival (OS) in this group remains <1 year 19. Gastric cancer is a heterogeneous disease that demands continued attention and research with regard to prevention, early detection and novel therapeutic options. Therefore, finding specific markers for early diagnosis of this cancer is very important in early treatment and preventing more cancer progress.

The human HER-3 is detected in tissues such as brain, prostate, mammary gland, lung, spinal cord, kidney, and liver 13. There are increasing reports for HER-3 role in onset and development of cancer. HER-3 can become activated with direct binding of ligands such as Neuregukin-1 (NRG-1) and Neuregulin-2 (NRG-2), but its intrinsic kinase domain is deficient and it is therefore unable to trigger downstream signaling pathways on its own 20–22. Thus, HER-3 receptor should dimerize with other ErbB receptors, especially with HER-2, to drive downstream signaling pathways. One of the requisites for tyrosine kinase activity of HER-2 is the contribution of HER-3 receptor 14,22. Several signaling pathways can be activated by hetero-dimerization of HER-3 with other members of ErbB family including PI3K/Akt, PLCγ/PKC, Jac/Stat and MEK/ MAPK. Of all dimers formed by the members of ErbB receptor family, HER-2/HER-3 heterodimers are the most potent activator of downstream signaling 14,22. Interestingly, the cells that lack HER-3 receptors or only a few of them, cannot proliferate and these anti-proliferative properties resulted from loss of HER-3 could not be compensated by HER-1 or HER-4 partners, which suggests the great importance of HER-3 in carcinogenesis 10. HER-2/HER-3 heterodimers induce cell proliferation and inhibit apoptosis mainly by enhancing PI3K/Akt signaling pathway. The PI3K/Akt pathway is involved in survival and activated in many cancers. In fact, direct binding of HER-3 to PI3K enhances PI3K/Akt pathway induced by HER-2/HER-3 heterodimers 10,20,23. Recently, the role of HER-3 in primary tumors and acquired resistance of HER-2+ breast cancer cells to chemo drug therapy has attracted serious attentions 24. HER-3, as a major cause of treatment failure in chemotherapy, mainly confers its influences through activation of PI3K/Akt signaling pathway 25.

Several oncogenic mutations in *HER-3* gene have been detected in gastric and colon cancers that elucidate the role of *HER-3* in development of cancer 26. Jeong *et al* detected protein- altering mutations in HER-3 in 1% of patients with colon cancer 27. Jaiswal *et al* identified *HER-3* somatic mutations in 11% of patients with colon cancer and 12% patients with gastric cancer 12. Wang *et al* reported frequent mutations in the *HER-3* gene in 10% of patients with gastric cancer 28. Based on a meta-analysis by Chen *et al*, *HER-3* plays an essential role in the clinicopathology and prognosis of gastric cancer 29.

All the above mentioned observations suggest that *HER-3* is the most important partner of *HER-2* in *HER-2* positive breast cancer and greater attentions must be paid on its molecular functions in chemo-resistance and carcinogenesis. Since, the overexpression of *HER-3* has been observed in some cancers such as breast and gastric adenocarcinoma, in this study, the expression level of *HER-3* was evaluated in Iranian patients with gastric adenocarcinoma. Obtained data in this study demonstrated that the expression of *HER-3* gene is elevated in gastric tumors compared to non-tumor tissues. Notably, the average expression level of *HER-3* gene was higher in T3–T4 classified tumors. Also, *HER-3* knocking down by siRNA reduced cell viability and accumulated cells in G2/M phase of cell cycle.

Osaki *et al* and Zhang *et al* showed that *HER-3* causes uncontrolled cell cycle by PI3K/Akt signaling pathway 30,31. In our results, G2/M arrest occurred in MKN45 cells by silencing of *HER-3*. Accumulation of cells in G2/M phase by *HER-3* siRNA is probably related to the Akt inactivation by decreasing cyclin B1 that is resulted in cell proliferation inhibition.

Our results confirmed Wu *et al*’s results that showed *HER-3* is involved in cell proliferation and viability in gastric cancer cell lines 32. Taken together, these findings suggest a potential correlation between HER-3 expression level and gastric tumor progression and malignancy.

## Conclusion

Our study demonstrates the importance of expression level of *HER-3* member for gastric cancer diagnosis and prognosis. This study might provide further insight into the prevention and detection of gastric cancer in early stages in near future.

## References

[1] Center MM Jemal A Ward E. International trends in colorectal cancer incidence rates. Cancer Epidemiol Biomarkers Prev 2009;18(6):1688–1694. 19505900 10.1158/1055-9965.EPI-09-0090

[2] Ferlay J Shin HR Bray F Forman D Mathers C Parkin DM. Estimates of worldwide burden of cancer in 2008: GLOBOCAN 2008. Int J Cancer 2010;127(12):2893–2917. 21351269 10.1002/ijc.25516

[3] Hu B El Hajj N Sittler S Lammert N Barnes R Meloni-Ehrig A. Gastric cancer: classification, histology and application of molecular pathology. J Gastrointest Oncol 2012;3(3):251–261. 22943016 10.3978/j.issn.2078-6891.2012.021 PMC3418539

[4] Xia W Lau YK Zhang HZ Xiao FY Johnston DA Liu AR Combination of EGFR, HER-2/neu, and HER-3 is a stronger predictor for the outcome of oral squamous cell carcinoma than any individual family members. Clin Cancer Res 1999;5(12):4164–4174. 10632356

[5] Hubbard SR Till JH. Protein tyrosine kinase structure and function. Annu Rev Biochem 2000;69:373–398. 10966463 10.1146/annurev.biochem.69.1.373

[6] Klapper LN Kirschbaum MH Seta M Yarden Y. Biochemical and clinical implications of the ErbB/HER signaling network of growth factors. Adv Cancer Res 1999; 77:25–79. 10549355

[7] Mendelsohn J Baselga J. The EGF receptor family as targets for cancer therapy. Oncogene 2000;19(56):6550–6565. 11426640 10.1038/sj.onc.1204082

[8] Atalay G Cardoso F Awada A Piccart MJ. Novel therapeutic strategies targeting the epidermal growth factor receptor (EGFR) family and its downstream effectors in breast cancer. Ann Oncol 2003;14(9):1346–1363. 12954573 10.1093/annonc/mdg365

[9] Lemmon MA Schlessinger J. Regulation of signal transduction and signal diversity by receptor oligomerization. Trends Biochem Sci 1994;19(11):459–463. 7855887 10.1016/0968-0004(94)90130-9

[10] Holbro T Civenni G Hynes NE. The ErbB receptors and their role in cancer progression. Expe Cell Res 2003;284(1):99–110. 10.1016/s0014-4827(02)00099-x 12648469

[11] Amin DN Sergina N Ahuja D McMahon M Blair JA Wang D Resiliency and vulnerability in the HER2-HER3 tumorigenic driver. Sci Transl Med 2010;2(16):16ra7. 10.1126/scitranslmed.3000389 PMC3033659 20371474

[12] Jaiswal BS Kljavin NM Stawiski EW Chan E Parikh C Durinck S Oncogenic ERBB3 mutations in human cancers. Cancer Cell 2013;23(5):603–617. 23680147 10.1016/j.ccr.2013.04.012

[13] Sithanandam G Anderson LM. The ERBB3 receptor in cancer and cancer gene therapy. Cancer Gene Ther 2008; 15(7):413–448. 18404164 10.1038/cgt.2008.15 PMC2761714

[14] Ma J Lyu H Huang J Liu B. Targeting of erbB3 receptor to overcome resistance in cancer treatment. Mol Cancer 2014;13:105. 24886126 10.1186/1476-4598-13-105 PMC4022415

[15] Citri A Skaria KB Yarden Y. The deaf and the dumb: the biology of ErbB-2 and ErbB-3. Exp Cell Res 2003; 284(1):54–65. 12648465 10.1016/s0014-4827(02)00101-5

[16] McDonagh CF Huhalov A Harms BD Adams S Paragas V Oyama S Antitumor activity of a novel bispecific antibody that targets the ErbB2/ErbB3 oncogenic unit and inhibits heregulin-induced activation of ErbB3. Mol Cancer Ther 2012;11(3):582–593. 22248472 10.1158/1535-7163.MCT-11-0820

[17] Lee Y Ma J Lyu H Huang J Kim A Liu B. Role of erbB3 receptors in cancer therapeutic resistance. Acta Biochim Biophys Sin (Shanghal) 2014;46(3):190–198. 10.1093/abbs/gmt150 24449784

[18] Carcas LP. Gastric cancer review. J Carcinog 2014;13: 14. 25589897 10.4103/1477-3163.146506 PMC4278094

[19] Cervantes A Roda D Tarazona N Roselló S Pérez-Fid-algo JA. Current questions for the treatment of advanced gastric cancer. Cancer Treat Rev 2013;39(1):60–67. 23102520 10.1016/j.ctrv.2012.09.007

[20] Stern DF. ERBB3/HER3 and ERBB2/HER2 duet in mammary development and breast cancer. J Mammary Gland Biol Neoplasia 2008;13(2):215–223. 18454306 10.1007/s10911-008-9083-7 PMC6590701

[21] Hsieh AC Moasser MM. Targeting HER proteins in cancer therapy and the role of the non-target HER3. Br J Cancer 2007;97(4):453–457. 17667926 10.1038/sj.bjc.6603910 PMC2360352

[22] Koutras AK Fountzilas G Kalogeras KT Starakis I Iconomou G Kalofonos HP. The upgraded role of HER3 and HER4 receptors in breast cancer. Crit Rev Oncol Hematol 2010;74(2):73–78. 19481955 10.1016/j.critrevonc.2009.04.011

[23] Green AR Barros FF Abdel-Fatah TM Moseley P Nolan CC Durham AC HER2/HER3 heterodimers and p21 expression are capable of predicting adjuvant trastuzumab response in HER2+ breast cancer. Breast Cancer Res Treat 2014;145(1):33–44. 24706169 10.1007/s10549-014-2925-7 PMC3984415

[24] Seruga B Tannock IF. Chemotherapy-based treatment for castration-resistant prostate cancer. J Clin Oncol 2011;29(27):3686–3694. 21844499 10.1200/JCO.2010.34.3996

[25] Jathal MK Chen L Mudryj M Ghosh PM. Targeting ErbB3: the new RTK (id) on the prostate cancer block. Immunol Endocr Metab Agents Med Chem 2011;11(2):131–149. 21603064 10.2174/187152211795495643 PMC3095967

[26] Wang Y Yang H Duan G. HER3 over-expression and overall survival in gastrointestinal cancers. Oncotarget 2015;6(40):42868–42878. 26517355 10.18632/oncotarget.5998 PMC4767477

[27] Jeong EG Soung YH Lee JW Lee SH Nam SW Lee JY ERBB3 kinase domain mutations are rare in lung, breast and colon carcinomas. Int J Cancer 2006;119(12):2986–2987. 16998794 10.1002/ijc.22257

[28] Wang K Kan J Yuen ST Shi ST Chu KM Law S Exome sequencing identifies frequent mutation of ARI-D1A in molecular subtypes of gastric cancer. Nat Genet 2011;43(12):1219–1223. 22037554 10.1038/ng.982

[29] Cao GD Chen K Xiong MM Chen B. HER3, but not HER4, plays an essential role in the cinicopathology and prognosis of gastric cancer: a meta-analysis. PLoS One 2016;11(8):e0161219. 10.1371/journal.pone.0161219 PMC4990181 27536774

[30] Osaki M Oshimura M Ito H. PI3K-Akt pathway: its functions and alterations in human cancer. Apoptosis 2004;9(6):667–676. 15505410 10.1023/B:APPT.0000045801.15585.dd

[31] Zhang Y Gonzalez RM Zangar RC. Protein secretion in human mammary epithelial cells following HER1 receptor activation: influence of HER2 and HER3 expression. BMC Cancer 2011;11:69. 21320340 10.1186/1471-2407-11-69 PMC3050851

[32] Wu X Chen Y Li G Xia L Gu R Wen X Her3 is associated with poor survival of gastric adenocarcinoma: Her3 promotes proliferation, survival and migration of human gastric cancer mediated by PI3K/AKT signaling pathway. Med Oncol 2014;31(4):903. 24623015 10.1007/s12032-014-0903-x

